# Transmating: conjugative transfer of a new broad host range expression vector to various *Bacillus* species using a single protocol

**DOI:** 10.1186/s12866-018-1198-4

**Published:** 2018-06-08

**Authors:** Simon Heinze, Petra Kornberger, Christian Grätz, Wolfgang H. Schwarz, Vladimir V. Zverlov, Wolfgang Liebl

**Affiliations:** 1 Department of Microbiology, Technical University of Munich, Emil-Ramann-Str. 4, D-85354 Freising-Weihenstephan, Germany; 2 Institute of Molecular Genetics, Russian Academy of Science, Kurchatov Sq. 2, 123182 Moscow, Russia

**Keywords:** Triparental conjugation, Broad host range, Shuttle vector, sfGFP, *Bacillus*, *Paenibacillus*, Heterologous expression, Genetic modification, Plasmid transfer, Transmating

## Abstract

**Background:**

The genus *Bacillus* includes a great variety of species with potential applications in biotechnology. While species such as *B. subtilis* or *B. licheniformis* are well-known and used to provide various products at industrial scale, other *Bacillus* species are less characterized and are not yet used in commercial processes. One reason for this is the fact that genetic manipulation of new isolates is usually complicated with conventional techniques which have to be adapted to each new strain. Even in well-established strains, the available transformation protocols often suffer from low efficiencies.

**Results:**

In this paper, we provide a new broad host range *E. coli*/*Bacillus* shuttle vector, named pBACOV (***B****acillus* **co**njugation **v**ector), that can be efficiently transferred to various *Bacillus* species using a single protocol. A variant of pBACOV carrying the *sfGFP* gene was successfully transferred to eight different species from the genus *Bacillus* and to one *Paenibacillus* species using triparental conjugation (“transmating”). This was achieved using a single protocol and worked for nine out of eleven tested acceptor species. The transmating procedure was used to test expression of the heterologous reporter gene *sfGFP* under control of the P_aprE_-promoter from *B. subtilis* in several *Bacillus* species in parallel. Expression of *sfGFP* was found in eight out of nine transmates. For several of the tested species, this is the first report of a method for genetic modification and heterologous gene expression. The expression level, analyzed by measuring the relative sfGFP-fluorescence normalized to the cell density of the cultures, was highest in *B. mojavensis*.

**Conclusions:**

The new shuttle vector pBACOV can be transferred to many different *Bacillus* and *Paenibacillus* species using a simple and efficient transmating protocol. It is a versatile tool facilitating the application of recombinant DNA technology in new as well as established strains, or selection of an ideal host for heterologous gene expression from a multitude of strains. This paves the way for the genetic modification and biotechnological exploitation of the broad diversity of species of *Bacillus* and related genera as well as different strains from these species.

**Electronic supplementary material:**

The online version of this article (10.1186/s12866-018-1198-4) contains supplementary material, which is available to authorized users.

## Background

*Bacillus* species are among the most extensively examined bacteria and are used for a variety of applications. Most prominent among them is *Bacillus subtilis*, which was the first *Bacillus* strain successfully transformed with purified DNA [1]. Today, *B. subtilis* and other members of the genus are major workhorses in industrial microbiology, mainly due to their ability to secrete large quantities of extracellular enzymes [2]. While species such as *B. subtilis* and *B. licheniformis* have already been isolated in the nineteenth century and are therefore well-known and widely applied, new species and strains continue to be isolated. Some of these new isolates have interesting properties for potential applications, but are not yet widely used. Additional File 1 gives a non-exhaustive overview of *Bacillus* strains which are already applied for the production of enzymes and metabolites, as well as rather “exotic” strains which are not yet applied but show promising characteristics. The listed species were also used in this study.

Recombinant DNA technology can greatly improve the performance of microorganisms for selected applications. Although *Bacillus* species exhibit promising characteristics, currently the Gram-negative *Escherichia coli* is the most widely used host for heterologous gene expression, especially in the context of metabolic engineering and production of small molecules. This is due to the fact that efficient genetic manipulation techniques and system-level strategies exist for this microorganism [3]. While efficient transformation protocols and methods for manipulation of the chromosome are available for *B. subtilis,* it is more challenging to perform such experiments with sufficient efficiency in other *Bacillus* hosts, especially in wild-type strains which are difficult to modify genetically [2, 4]*.* One reason for this is that transformation of bacteria with plasmid DNA generally requires specified protocols and adaptation of the procedures for each strain.

Common methods to introduce recombinant DNA are the use of natural competence in case of *B. subtilis*, protoplast transformation (often used for *B. megaterium*)*,* electroporation, and mobilization of plasmids from *E. coli* to Gram-positive recipients by conjugation. In most cases, the elaborate preparation of naturally or otherwise competent cells is required and usually, different methods are needed for different strains. In addition to the fact that the available methods are usually specific for one strain, they also have other drawbacks: protoplasts are difficult to handle and do not survive freezing; natural competence is observed in *B. subtilis* but not in most other *Bacillus* species and the efficiency is rather low; electroporation is an efficient method, but requires individual determination of ideal parameters such as medium composition and electric field strength [5–7]. In addition, it can be troublesome to transfer previously established genetic transformation methods (such as chemical/heat transformation, protoplast transformation or electroporation) even between strains of the same species, due to differences e.g. in special medium requirements (for example regeneration media after protoplast transformation) or differences in their restriction/modification systems.

Methods for the efficient introduction of genes into multiple species and strains of bacilli without the need to adapt the protocol to each strain of interest are currently not available, but would be of high value, for example to enable fast examination which strain or species is best suited for expression of a given gene.

## Results

**Fig. 1:**
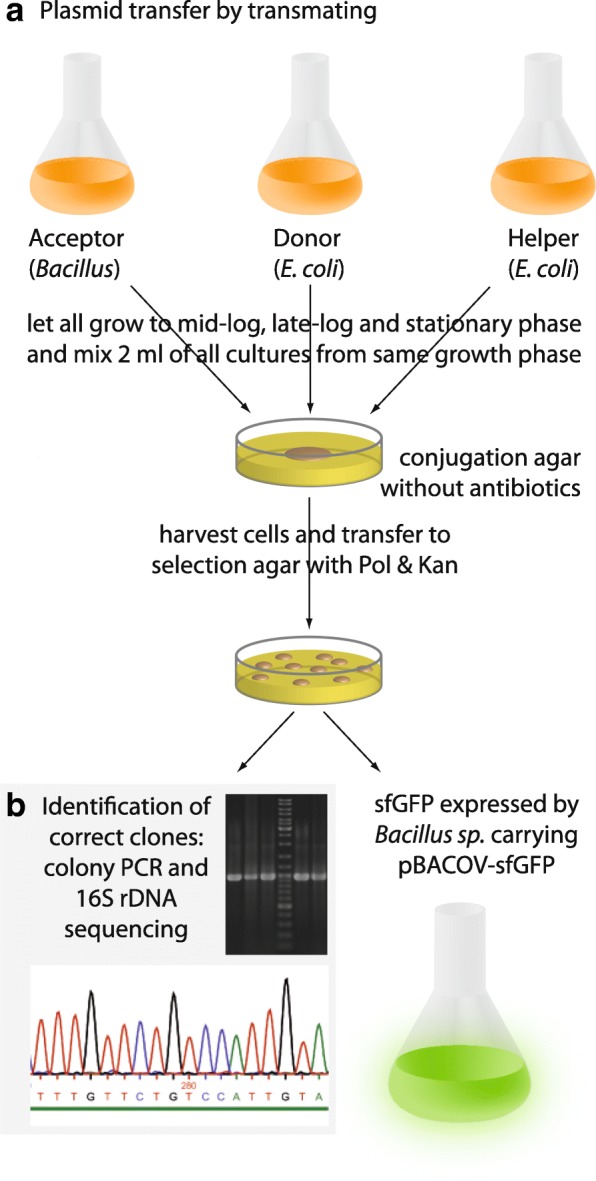
Steps for transfer of pBACOV-sfGFP from *E. coli* to *Bacillus* species by transmating. Triparental conjugation (transmating) requires an acceptor strain (strain of a *Bacillus* species of choice), a donor strain (e. g. *E. coli* TOP10 carrying pBACOV-sfGFP) and a helper strain (*E. coli* HB101 pRK2013). After conjugation on agar plates without selection, the cells are transferred to appropriate antibiotic-containing plates (Pol concentration < MIC of acceptor, Kan concentration > MIC of acceptor) for selection of transmates (**a**). Plasmid content and the identity of the host strain were confirmed by colony PCR and 16S rRNA gene sequencing (**b**) before further characterization of the transmates (**c**). MIC: minimal inhibitory concentration, Kan: kanamycin, Pol: polymyxin B

To enable fast evaluation of the suitability of multiple bacterial species for heterologous gene expression and to assess their genetic accessibility, we established a new broad-host range shuttle vector in combination with a simple procedure for plasmid transfer by triparental conjugation. The method, named “transmating”, should be suited for a broad variety of strains without the need of protocol adaptation for the individual strains. A schematic overview of the method is given in Fig. 1.

### Design of pBACOV

The newly developed plasmid pBACOV (***Ba****cillus* **co**njugation **v**ector) includes replication origins and selection markers for *E. coli* and *Bacillus* as well as the expression cassette from pBE-S and the RK2 conjugation origin (*oriT*/*traJ*) from plasmid pKVM4, a mobilizable plasmid used to generate markerless gene deletions in *B. licheniformis* [8]. The expression cassette contains the *B. subtilis* promoter P_aprE_, the *aprE* signal peptide for secretory protein expression, a multiple cloning site (MCS) and the coding sequence for a C-terminal hexahistidine tag (His_6_-tag) for protein purification (see Materials and Methods).

**Fig. 2:**
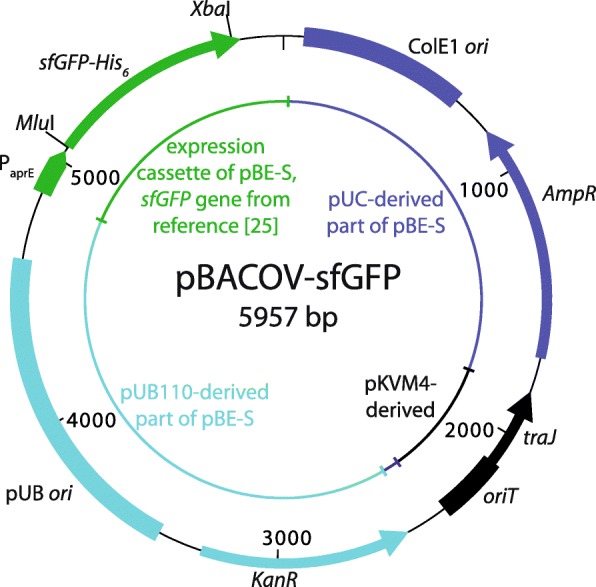
Schematic map of the shuttle vector pBACOV with the inserted target gene sfGFP. ColE1 *ori*: origin of replication for *E. coli; AmpR*: Ampicillin resistance gene for selection in *E. coli*; *oriT*/*traJ*: origin of transfer for conjugative plasmid transfer; *KanR:* Kanamycin resistance gene for selection in *Bacillus*; pUB *ori*: origin of replication for *Bacillus*; P_aprE_: aprE promoter from *B. subtilis*; *sfGFP-His*_*6*_: target gene *sfGFP* fused with a sequence encoding a C-terminal His_6_-tag

To generate pBACOV-sfGFP (Fig. 2), the *sfGFP* gene coding for super-folder green fluorescent protein [9] was inserted into pBACOV, while removing the *aprE* signal peptide. Thus, sfGFP is expressed intracellularly, allowing to compare promoter strength between different strains containing pBACOV-sfGFP without having to be concerned about additional influences that secretion might have on the level of sfGFP produced. The expected plasmid sequences of pBACOV and pBACOV-sfGFP were confirmed by sequencing.

### Establishment of the minimal inhibitory concentrations of kanamycin and polymyxin B for all tested strains

The selection agar contained kanamycin (Kan) for the selection of colonies carrying the plasmid and polymyxin B (Pol) for counter-selection against the *E. coli* donor and helper strains. The antibiotic concentration in the selection agar must be adjusted such that the sensitivity toward Kan was high enough to enable selection for colonies carrying the plasmid, while sufficient resistance against Pol was necessary to ensure effective counter-selection against *E. coli*. The standard concentration of antibiotics in the selection agar was 10 μg/ml for Kan and 40 μg/ml for Pol. These concentrations were found to be suited for *B. licheniformis* DSM13, *B. pumilus* DSM27, *B. sonorensis* DSM13779 and *Paenibacillus polymyxa* DSM356. Other *Bacillus* species tested showed resistance to 10 μg/ml Kan or sensitivity to 40 μg/ml Pol. In these cases, the minimal inhibitory concentrations of Kan or Pol had to be established by serial dilution tests before transmating with pBACOV-sfGFP could be attempted. The results of the serial dilution tests are listed in Additional File 2. Based on these results, seven additional strains were selected for transmating experiments with adapted concentrations of the selection antibiotics: *B. mycoides* DSM2048*, B. megaterium* DSM32*, B. pseudomycoides* DSM12442 (concentrations used: Pol: 40 μg/ml, Kan: 50 μg/ml), *B. subtilis* RIK1285*, B. mojavensis* DSM9205*, B. vallismortis* DSM11031 (Pol: 10 μg/ml, Kan: 10 μg/ml) and *B. oleronius* WS8036 (Pol: 2.5 μg/ml, Kan: 10 μg/ml).

### Transmating pBACOV-sfGFP to different *Bacillus* species

Plasmid pBACOV-sfGFP, which was constructed as a derivative of vector pBACOV (see above and Fig. 2), was used to study (i) transfer to a large number of different *Bacillus* species by a single routine transmating procedure and (ii) the expression of the heterologous reporter gene *sfGFP* in all transmates.

The transfer of pBACOV-sfGFP from *E. coli* TOP10 to various *Bacillus* acceptor strains was achieved by transmating using *E. coli* HB101 pRK2013 as helper strain [10, 11] as described in Material & Methods. Plasmid pRK2013 is a self-transmissible plasmid (size: 48 kbp) containing the broad host range transfer system of RK2 [10, 12]. It is efficiently transferred between Gram-negative bacteria and can promote the conjugal transfer of unrelated plasmids [10, 12]. The mechanism for triparental plasmid transfer is thought to involve two steps: first, the helper plasmid pRK2013 is transferred to the donor strain which carries the mobilizable, but not self-transmissible, expression plasmid pBACOV. Now, the genes necessary for conjugation can be expressed in the donor strain and thus the expression plasmid pBACOV can be transferred to the acceptor strain. For each acceptor strain, three transmating batches were made. These contained either cells from the overnight pre-cultures, cells from the mid-exponential growth phase (OD_600_ between 0.6 and 0.9) or cells from the late exponential phase (OD_600_ ≥ 1.2). After incubation of the selection plates, colonies were counted and correct transmates were identified by analytical PCR using plasmid specific primers (pBACOV-seq_4894fw and pBACOV-Seq-rv). The 16S rRNA gene sequence of selected transmates was determined to verify that the clones obtained after selection/counter-selection belonged to the correct acceptor species. This was especially important for *B. mojavensis*, *B. vallismortis* and *B. oleronius*, i. e. the strains grown at reduced Pol concentrations, in order to eliminate the possibility of growth of *E. coli* under these milder counter-selection conditions. Additionally, special attention was paid to the colony morphology: to determine the transmating efficiency, only colonies with the morphology typical for the respective acceptor strains were considered. The Pol concentration of 10 μg/ml used for the matings with *B. mojavensis* and *B. vallismortis* could not inhibit growth of *E. coli* completely, since some of the tested colonies were *E. coli*, while others were *Bacillus* transmates. Here, the colony morphology was helpful for the identification of correct clones as a prescreening before 16S rRNA gene sequencing. In the case of *B. oleronius* all colonies chosen from the selection plates for 16S rRNA gene sequencing turned out to be *E. coli.* This indicates that although the minimal inhibitory Pol concentration for *E. coli* in liquid cultures was below 2.5 μg/ml, effective counter-selection against *E. coli* was not possible on agar plates at this concentration. Thus, transfer of pBACOV-sfGFP to *B. oleronius* was regarded as not successful.

Transfer of pBACOV-sfGFP to all other selected acceptor strains was successful with the only exception of *B. megaterium.* The total number of colonies on selection agar varied between the different species as well as the three transmating batches for each strain, but was never below 25 (for examples of yielded colony numbers, see Table 1). The most colonies were obtained with *P. polymyxa* DSM356 (total number of colonies: 1750) and *B. subtilis* RIK1285 (several thousand colonies, partially confluent growth on selection plates).

**Table 1:** Yield of colonies obtained in representative transmating experiments with each acceptor strain

Species	number of colonies with typical morphology
*B. subtilis*	> 5000 *
*P. polymyxa*	1750
*B. vallismortis*	849
*B. pumilus*	655
*B. mojavensis*	574
*B. sonorensis*	178
*B. licheniformis*	37
*B. mycoides*	n. q. **
*B. pseudomycoides*	n. q. **
*B. oleronius*	0
*B. megaterium*	0

Only colonies with the typical morphology of the respective acceptor strain were counted. On selection plates with polymyxin B concentrations ≤10 μg/ml, additional growth of *E. coli* was observed. *: for *B. subtilis*, several thousand distinct colonies and partially confluent growth were observed. **: for *B. mycoides* and *B. pseudomycoides*, the exact number of colonies could not be quantified (n.q.), due to confluent growth of large, mycelium-like colonies (diameter > 10 mm)

### Measurement of sfGFP fluorescence

**Fig. 3:**
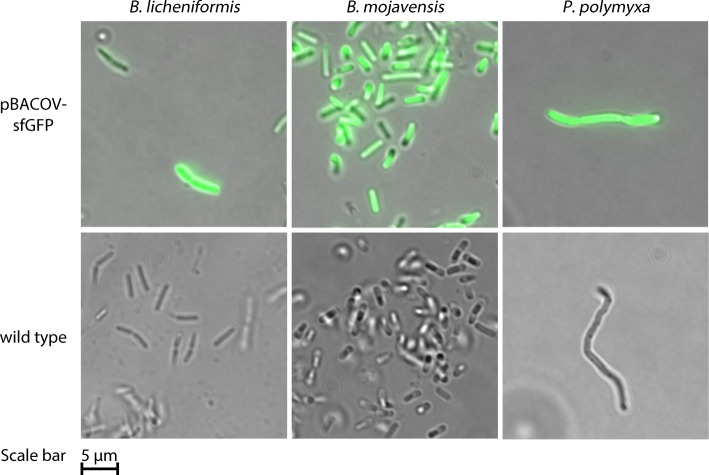
Visualization of sfGFP expression by fluorescence microscopy. Top row from left to right: Transmates of *B. licheniformis, B. mojavensis* and *P. polymyxa* carrying pBACOV-sfGFP. Bottom row from left to right: *B. licheniformis, B. mojavensis* and *P. polymyxa* wild type strains. The scale bar corresponds to 5 μm. Images were recorded using a Zeiss Axio Imager.M1 fluorescence microscope equipped with a Zeiss AxioCam MRm camera

Production of sfGFP by selected transmates was studied in order to demonstrate the usefulness of pBACOV as broad host range expression plasmid. Non-transmated cultures of the same species served as negative controls. SM-Cas medium was used, because autofluorescence was not detectable with this medium. After 24 h of incubation, the cultures were diluted to an OD_600_ of about 1 and the fluorescence was measured (excitation: 470 nm, emission: 520 nm). Table 2 summarizes the relative fluorescence normalized to the OD_600_ of the cultures (fluorescence intensity/OD_600_). In eight out of nine cultures of strains carrying pBACOV-sfGFP, sfGFP-fluorescence was detectable, which shows that the P_aprE_-promoter is functional in these bacteria. *B. sonorensis* was the only transmated species which did not show detectable sfGFP fluorescence*.* Hence, P_aprE_ can be used for the expression of heterologous genes in almost 90% (eight of nine) of the tested strains. The strongest relative fluorescence was observed with *B. mojavensis.* The sfGFP-fluorescence was also visualized by fluorescence microscopy. Images of *B. licheniformis, B. mojavensis* and *P. polymyxa* with or without pBACOV-sfGFP are shown in Fig. 3.

**Table 2:** sfGFP fluorescence of different bacterial species carrying pBACOV-sfGFP relative to *B. subtilis* RIK1285 pBACOV-sfGFP

Species	Relative fluorescence [%]	
	pBACOV-sfGFP	wild-type
*B. mojavensis*	383 ± 5.1	n. d.
*B. vallismortis*	239 ± 5.9	0.9 ± 0.1
*B. licheniformis*	160 ± 8.8	n. d.
*B. subtilis*	100 ± 0.6	n. d.
*B. pumilus*	56.1 ± 0.9	n. d. *
*B. pseudomycoides*	41.0 ± 1.5	n. d.
*P. polymyxa*	16.7 ± 0.5	3.4 ± 0.2
*B. mycoides*	14.9 ± 0.1	3.1 ± 0.0
*B. sonorensis*	n. d.	1.1 ± 0.1

Strains were grown for 24 h at 37 °C, 180 rpm (*B. mycoides*: 72 h at 30 °C, 180 rpm) in SM-Cas medium with supplements, if appropriate. For details, see Material & Methods. Fluorescence was detected with cultures diluted to OD_600_ = 1 and the fluorescence intensity (excitation: 470 nm, emission 520 nm) and the OD_600_ were determined. The relative fluorescence (fluorescence intensity/OD_600_) was calculated by normalizing the measured fluorescence intensity by the OD_600_. The relative fluorescence of *B. subtilis* RIK1285 pBACOV-sfGFP was defined as 100%. The given values are mean values ± standard deviation of triplicate measurements. The asterisk * indicates that the *B. pumilus* wild-type culture did not reach an OD_600_ of 1 within the 24 h of incubation and therefore was not diluted for fluorescence measurements. n. d.: not detectable (defined as relative fluorescence < 0.7%)

## Discussion

Conjugal transfer of plasmid DNA from Gram-negative *E. coli* to Gram-positive bacteria has been reported before [13, 14], including *B. licheniformis* and thermophilic *Bacillus* strains [4, 14, 15], but no comprehensive study exists showing the versatility of this gene transfer method to introduce plasmids into various different species of mesophilic bacilli. Plasmid pBACOV, introduced in this work, is a new broad host range vector which can easily be transferred to an acceptor strain of choice using triparental conjugation (“transmating”) and can be used for heterologous gene expression in various *Bacillales* species. pBACOV-sfGFP, containing *sfGFP* as reporter gene, was successfully transferred to strains of widely used and well-studied species such as *B. licheniformis* or *B. subtilis*, but also to more “exotic” species. To our knowledge, this is the first report of recombinant DNA technology applied in *B. sonorensis, B. mycoides, B. pseudomycoides, B. vallismortis* and *B. mojavensis*, thus making these species accessible for genetic engineering*.* Since *Bacillus* species in general have high potential for biotechnological applications, there is a great interest in expanding the number of genetically accessible strains. Usage of the pBACOV vector in combination with the transmating protocol described herein can pave the way to future applications.

In this study, we strived to test the applicability of pBACOV and our transmating method in as many species as possible from the genus *Bacillus* and closely related genera. While the majority of the tested strains belonged to the genus *Bacillus*, pBACOV also worked in the genus *Paenibacillus*, as was demonstrated with *P. polymyxa* DSM356. The pUB origin of replication stems from *Staphylococcus aureus* and is known to be active in a wide range of low GC Gram-positive bacteria (*Firmicutes*), including anaerobic *Clostridia* [16, 17]. Therefore it is likely that pBACOV can be transferred to even more genera.

In this study we did not aim to optimize the frequency of transfer between the *E. coli* donor and the acceptor bacilli but rather wanted to develop a simple, versatile method that can be applied to many strains without extensive adaptation to the different acceptor strains. In our hands, transmates were obtained for nine of the eleven *Bacillus* and *Paenibacillus* species tested. Transmating failed in only two of the cases tested, i.e. for *B. megaterium* DSM32 and *B. oleronius* WS8036. Regarding *B. megaterium*, the reason for the failed transmating is not known, but the cell envelope composition [18] or other special features may play a role.

To express heterologous target genes, pBACOV features the P_aprE_ promoter from *B. subtilis* [19–22]. We demonstrate that P_aprE_ is functional in seven additional *Bacillus* species using *sfGFP* as a reporter gene and report for the first time heterologous gene expression in four new *Bacillus* species (*B. mojavensis, B. mycoides, B. pseudomycoides* and *B. vallismortis*). The observed *sfGFP* expression levels ranged between about 0.15- and 3.8-fold of the value measured in *B. subtilis*. The reasons for the up to 25-fold differences in the apparent *sfGFP* expression levels found between different host strains are currently unknown, but various differences between the hosts’ gene expression machineries (differences in promoter P_aprE_ recognition, mRNA stability, sfGFP folding efficiency etc.) and also differences in the plasmid’s copy number may contribute to this observation.

It is possible to obtain large numbers of colonies with some strains (e.g. *P. polymyxa* DSM356, *B. subtilis RIK1285*). This makes the transmating system suitable for the screening of libraries, e. g. signal peptide libraries, in some of the new host strains from this work for optimizing the expression of a secretory protein of interest. This holds true even for species where fewer colonies were obtained, as the procedure can be easily scaled up. This is a big advantage over transformation procedures using purified plasmid DNA, where purification of large quantities of plasmid DNA is needed.

One limitation of our system is that sensitivity to Kan and resistance to Pol are prerequisites for the acceptor strain. Of the 24 bacteria included in this study, only 10 (approx. 42%) showed the necessary characteristics. The selection for transmates relies on Kan resistance conferred by the plasmid. By replacing the Kan resistance cassette of pBACOV with a different selection marker (such as chloramphenicol or tetracycline resistance), or by equipping the vector with two or even three selection markers, it should be possible to increase the versatility of pBACOV. For counter-selection against the *E. coli* donor and helper strains, the minimal inhibitory concentration of Pol is critical. In our experience, a concentration of at least 10 μg/ml Pol is required for sufficient counter-selection. At the attempt to transfer pBACOV-sfGFP to *B. oleronius* at a Pol concentration of 2.5 μg/ml, a substantial number of *E. coli* colonies was detected on the selection agar. In some instances, colonies of *E. coli* even grew at 10 μg/ml, resulting in false-positive clones which had to be eliminated via rigorous discriminating tests. In this work we used assessment of the characteristic colony morphology and 16S rRNA gene sequencing for the unequivocal identification of the *B. mojavensis* and *B. vallismortis* transmates, but other methods, including Gram-staining or differentiating physiological tests may also be useful to discriminate between real acceptor strain transmates and false-positive *E. coli* clones. Alternative options for effective counter-selection against *E. coli* could include the use of spontaneous antibiotic-resistant mutants of the acceptor strain or culture media which inhibit growth of *E. coli,* such as MRS agar [23].

## Conclusions

This study introduces a universal, simple and efficient method for the transfer of plasmid DNA into a large number of different *Bacillus* and *Paenibacillus* species. We stress that the same procedure worked for most tested strains without the need for individual optimization. The use of pBACOV enabled for the first time the demonstration of heterologous gene expression in *B. mojavensis, B. mycoides, B. pseudomycoides,* and *B. vallismortis.* Its expression promoter P_aprE_ from *B. subtilis* was functional in eight out of nine transmated species.

The new plasmid pBACOV in combination with the transmating protocol facilitates application of recombinant DNA technology in new as well as established *Bacillus* and *Paenibacillus* strains. It can aid in the selection of the best-suited host for heterologous gene expression from a large number of candidate strains, thereby enabling the research community to tap into the abundant source of hitherto unused *Bacillus* species with potential for application in biotechnology.

## Methods

### Bacterial strains and growth conditions:

The bacterial strains used in this study are listed in Additional File 2. All *Bacillus* and *Paenibacillus* strains were obtained from DSMZ (Leibniz-Institut DSMZ-Deutsche Sammlung von Mikroorganismen und Zellkulturen GmbH, Braunschweig, Germany), except *B. bataviensis* WS4576, *B. ginsengihumi* WS8095, *B. horikoshii* WS2157, *B. niabensis* WS9147, *B. niacini* WS4575, *B. oleronius* WS8036, *Fictibacillus arsenicus* WS4538 (obtained from the strain collection of the Chair for Microbial Ecology, Department of Biosciences, Technical University of Munich, Freising, Germany) and *B. subtilis* RIK1285 (Takara-Bio Inc., Kusatsu, Japan). *E. coli* TOP10 (Life technologies, Carlsbad, USA) was used for cloning and as donor strain in transmating experiments. *E. coli* HB101 carrying the plasmid pRK2013 (DSMZ 5599) [10, 11] was used as helper strain for transmating experiments. All strains were routinely grown in LB liquid medium [24] or agar plates at 37 °C (liquid cultures were shaken at 180 rpm), except for *B. mycoides*, which was grown at 30 °C. *E. coli* HB101 pRK2013 was grown with 50 μg/ml kanamycin (AppliChem GmbH, Darmstadt, Germany) and *E. coli* TOP10 carrying pBACOV or pBACOV-sfGFP was grown with 100 μg/ml carbenicillin (Carl Roth GmbH, Karlsruhe, Germany). *Bacillus* transmates were streaked on LB selection agar containing polymyxin B (Carl Roth GmbH) and Kan (for the applied concentrations see below, section “plasmid transfer by transmating”) or grown in liquid LB medium with Kan (same concentrations as the selection agar).

### Construction of pBACOV and pBACOV-sfGFP

To construct pBACOV, Gibson Assembly of three overlapping DNA fragments was applied [25], using the commercially available pBE-S plasmid (Takara-Bio Inc., size: 5938 bp) as chassis. pBE-S is composed of a fragment of the pUC plasmid, comprising the ColE1 origin of replication and ampicillin resistance gene (bp 1645–3556 of pBE-S) for propagation in *E. coli*, and a piece of plasmid pUB110, including the pUB origin of replication and the kanamycin resistance gene for *Bacillus* (bp 3557–5936 of pBE-S). Furthermore, an expression cassette is present after the pUB *ori* (bp 5937–517 of pBE-S). The expression cassette consists of the *B. subtilis* promoter P_aprE_, the *aprE* signal peptide for secretory protein expression, a multiple cloning site (MCS) and the coding sequence for a C-terminal hexahistidine tag (His_6_-tag) for protein purification. A stem-loop was predicted from bp 486 to 519, followed by a stretch of five T nucleotides, constituting a potential rho-independent transcriptional terminator. For construction of pBACOV, (i) an un-annotated segment of pBE-S (bp 580–1642 of pBE-S) was removed and (ii) a *oriT*/*traJ* region necessary for conjugative plasmid transfer was inserted between the antibiotic resistance genes.

Fragment 1 for Gibson Assembly (i. e. the pUB110-derived region and expression cassette, bp 3453–557 of pBE-S) was created using the primers pUB110_backbone_fw and pUB110_backbone_rv with pBE-S as template. Fragment 2 (the pUC-derived portion of pBE-S, bp 1642–3452) was amplified using the primers Fw_pUC_for_Gibson and Rv_pUC_from_pBE-S and pBE-S as template. Fragment 3 (*oriT/traJ*) was amplified by using the primers oriT_traJ_fw and oriT_traJ_rv and the plasmid pKVM4 as template [8]. To maintain compatibility of pBACOV with the signal peptide library from the *B. subtilis* Secretory Protein Expression System Kit (Takara-Bio Inc.), the *Eag*I restriction site within the *traJ* gene was removed by two-step QuikChange mutagenesis [26] using primers QC_g2015t_1 and QC_g2015t_2. The incorporated mutation (g2015t) is silent, exchanging the original alanine-codon GCC to GCA while removing the *Eag*I restriction site (CGGCCG is mutated to CTGCCG). For construction of pBACOV-sfGFP, the *sfGFP*-gene was amplified using the primers sfGFP-fw and sfGFP-rv and the plasmid pET28A-sfGFP (kindly provided by Mark Teese) as template. The sfGFP coding sequence corresponds to the sequences published in [27]. pBACOV was linearized using the restriction endonucleases *Mlu*I and *Xba*I, thereby removing the coding sequence of the aprE signal peptide. *sfGFP* was inserted into pBACOV using Gibson Assembly. All primers used are listed in Table 3.

**Table 3:** Primers used in this study

Primer Name	Sequence 5′ – 3′
pUB110_backbone_fw	GTCTAAGAAACCATTATTATCATGAC
pUB110_backbone_rv	CATAATAGTTATGCAGTTTGTAGAATGC
Fw_pUC_for_Gibson	CAAACTGCATAACTATTATGTACGAGCAAAAGGCCAGC
Rv_pUC_from_pBE-S	GTCAGGTGGCACTTTTCG
oriT_traJ_fw	TCCCCGAAAAGTGCCACCTGACTTAGTGCTTTACGGCACCTCG
oriT_traJ_rv	CATGATAATAATGGTTTCTTAGACGCTTCGGGGTCATTATAGCG
sfGFP-fw	TAAGCAAAAGGAGAGGGACGCGTATGAGCAAAGGTGAAGAACTG
sfGFP-rv	CATTAGTGGTGATGATGGTGATGTCTAGATTTATACAGTTCATCCATACCATG
pBACOV-seq_4894fw	CGAGTCTCTACGGAAATAGC
pBACOV-Seq-rv	CGATGAGCGCATTGTTAG
QC_g2015t_1	GCGGCGGCGGCAGGCATGAGCCTG
QC_g2015t_2	CAGGCTCATGCCTGCCGCCGCCGC

The vector sequences of pBACOV (accession number: MG599120.1) and pBACOV-sfGFP (accession number:MG599121.1) were deposited at GenBank.

### Determination of minimal inhibitory concentrations of kanamycin and polymyxin B

A single colony of the respective strain was carefully resuspended in 20 ml LB medium. Four glass test tubes were each filled with 2.5 ml of this suspension while a fifth glass test tube was filled with 5 ml. The 5 ml culture was supplemented with Kan (final concentration: 200 μg/ml) or Pol (final concentration: 40 μg/ml). Then, a dilution series was made by transferring 2.5 ml from one tube to the next, which resulted in a 2-fold dilution of the antibiotic per step (from 200 μg/ml to 12.5 μg/ml Kan; from 40 μg/ml to 2.5 μg/ml Pol). The tubes were incubated overnight at 180 rpm at the growth temperature of the respective test strain (see above). The minimal inhibitory concentration (MIC) was defined as the lowest concentration at which no growth was detectable.

### Plasmid transfer by transmating

To prepare pre-cultures for transmating experiments, 100 ml Erlenmeyer flasks containing 20 ml LB medium (supplemented with antibiotics if appropriate) were inoculated with single colonies of the *Bacillus* acceptor strain(s), the donor strain (*E. coli* TOP10 pBACOV-sfGFP) and the helper strain (*E. coli* HB101 pRK2013), respectively. The pre-cultures were incubated overnight as indicated above. The next day, the OD_600_ of all cultures was determined and the cultures were used to inoculate fresh flasks with 20 ml LB (supplemented with antibiotics if appropriate) to an OD_600_ of 0.2. The overnight pre-cultures were stored on ice for later use. The OD_600_ of the freshly inoculated flasks was checked regularly, starting 90 min after the beginning of incubation. Once the OD_600_ was between 0.6 and 0.9, 2 ml samples of each culture were taken and stored on ice. Incubation of the main cultures was continued until the OD_600_ was at least 1.2, at which point another round of 2 ml samples was collected and stored on ice. The collected donor, acceptor and helper samples of the same growth phase (overnight, OD_600_ between 0.6 and 0.9 or OD_600_ ≥ 1.2) were then mixed as follows: 2 ml of the donor cells were pelleted by centrifugation and the supernatant was discarded. All centrifugation steps were carried out at 1900 x g, 4 °C, for 5 min. The donor cell pellet was resuspended in 2 ml of acceptor culture and centrifuged again. The pellet was resuspended in 2 ml of the helper culture and centrifuged. The resulting pellet containing all three strains was washed twice with 1 ml of LB to remove traces of antibiotics. The cell pellet was resuspended in a small volume of remaining supernatant. Sterile cellulose acetate filters (pore size: 0.2 μm, diameter: 25 mm, Sartorius Stedim Biotech GmbH, Göttingen, Germany) were placed on LB agar plates and the mating mixtures were pipetted smoothly, with as little shearing as possible, to the middle of the filters. These conjugation plates were incubated overnight at 30 °C (three plates for each acceptor strain, each containing one mixture of donor/acceptor/helper cultures with the same OD_600_). The next day, the filters were carefully removed from the LB agar plates and placed into 5 ml Eppendorf reaction tubes (Eppendorf AG, Hamburg, Germany). The cells were harvested from the filters by adding 1 ml of LB medium and vortexing. These cell suspensions were then plated onto selection agar plates (LB agar containing Pol and Kan) and incubated overnight at 37 °C or for up to three days at 30 °C until colonies appeared. Routinely, the selection agar contained 10 μg/ml Kan and 40 μg/ml Pol. For *Bacillus* strains sensitive toward 40 μg/ml Pol or resistant against 10 μg/ml Kan, the respective antibiotic concentration was adapted based on the determined MIC as follows: 40 μg/ml Pol and 50 μg/ml Kan for *B. mycoides, B. megaterium* and *B. pseudomycoides*; 10 μg/ml Pol and 10 μg/ml Kan for *B. subtilis, B. mojavensis* and *B. vallismortis,* and 2.5 μg/ml Pol and 10 μg/ml Kan for *B. oleronius*. Correct transmates were identified by analytical PCR using two plasmid specific primers and by sequencing of the 16S rRNA gene using the primers 616 V and 630R [28].

### Expression of *sfGFP* and determination of relative fluorescence

SM-Cas medium, based on Spizizen’s Minimal Medium [1, 29], was used for *sfGFP-*expression experiments. The medium corresponds to the SM medium published online at *Subti*Wiki [30], with the addition of 1 g/l casamino acids but without tryptophane. The final composition of SM-Cas medium was 17.5 g/l K_2_HPO_4_, 7.5 g/l KH_2_PO_4_, 5 g/l glucose, 4 g/l l-glutamate, 2 g/l (NH_4_)_2_SO_4_, 1.26 g/l trisodium citrate dihydrate, 1 g/l casamino acids, 250 mg/l MgSO_4_, 5.5 mg/l CaCl_2_, 1 mg/l MnCl_2_∙4 H_2_O, 1.7 mg/l ZnCl_2_, 0.33 mg/l CuCl_2_∙2 H_2_O, 0.6 mg/l CoCl_2_∙6 H_2_O, 0.6 mg/l Na_2_MoO_4_∙2 H_2_O, 1.35 mg/l FeCl_3_∙6 H_2_O. For *P. polymyxa* DSM356, biotin was added to a final concentration of 150 μg/l and for the tryptophan auxotrophic *B. subtilis* RIK1285 tryptophan was added to a final concentration of 50 mg/l. 20 ml of SM-Cas (with Kan at appropriate concentrations) were inoculated with a single colony of the *Bacillus* transmates carrying pBACOV-sfGFP or the respective wild-type strains as negative controls and incubated at 37 °C, 180 rpm (*B. mycoides*: 30 °C). After 24 h (*B. mycoides*: 72 h), the OD_600_ of the cultures was measured. For determination of the relative fluorescence normalized to cell density, the cultures were diluted with SM-Cas to OD_600_ = 1. Next, the OD_600_ and the fluorescence intensity (excitation: 470 nm, emission: 520 nm) were measured using Uvette® cuvettes (Eppendorf) with a path length of 2 mm in a BioSpectrometer Fluorescence Photometer (Eppendorf). To calculate the relative fluorescence, the fluorescence intensity value was divided by the measured OD_600_, taking the path length of the cuvette into account. Each measurement was performed in triplicate.

## Additional files

Additional file 1: **Table 1.** Selection of well-known and exotic *Bacillus* species with some of their products and properties (including references). The listed species were also used in this study. (DOCX 20 kb)

Additional file 2: **Table 2.** Strains used in this study and determination of MIC of Kan or Pol (including references). (DOCX 19 kb)

## Abbreviations

Kan: Kanamycin
MIC: Minimal inhibitory concentration
OD_600_: Optical density at 600 nm
Pol: Polymyxin B
sfGFP: Super-folder green fluorescent protein

## References

[1] Spizizen J (1958). TRANSFORMATION OF BIOCHEMICALLY DEFICIENT STRAINS *OF BACILLUS SUBTILIS* BY DEOXYRIBONUCLEATE. Proc Natl Acad Sci U S A.

[2] Schallmey M, Singh A, Ward OP (2004). Developments in the use of *Bacillus* species for industrial production. Can J Microbiol.

[3] Liu L, Liu Y, Shin H-D, Chen RR, Wang NS, Li J (2013). Developing *Bacillus* spp. as a cell factory for production of microbial enzymes and industrially important biochemicals in the context of systems and synthetic biology. Appl Microbiol Biotechnol.

[4] Hertel R, Volland S, Liesegang H (2015). Conjugative reporter system for the use in *Bacillus licheniformis* and closely related *Bacilli*. Lett Appl Microbiol.

[5] Schumann W (2007). Production of recombinant proteins in *Bacillus subtilis*. Adv Appl Microbiol.

[6] Trieu-Cuot P, Carlier C, Martin P, Courvalin P (1987). Plasmid transfer by conjugation from *Escherichia coli* to gram-positive bacteria. FEMS Microbiol Lett.

[7] Xue G-P, Johnson JS, Dalrymple BP (1999). High osmolarity improves the electro-transformation efficiency of the gram-positive bacteria *Bacillus subtilis* and *Bacillus licheniformis*. J Microbiol Methods.

[8] Kostner D, Rachinger M, Liebl W, Ehrenreich A. Markerless deletion of putative alanine dehydrogenase genes in *Bacillus licheniformis* using a codBA-based counterselection technique. Microbiology. 2017; 10.1099/mic.0.000544. 10.1099/mic.0.000544 28984230

[9] Pédelacq J-D, Cabantous S, Tran T, Terwilliger TC, Waldo GS (2006). Engineering and characterization of a superfolder green fluorescent protein. Nat Biotechnol.

[10] Figurski DH, Helinski DR (1979). Replication of an origin-containing derivative of plasmid RK2 dependent on a plasmid function provided in trans. Proc Natl Acad Sci U S A.

[11] Ditta G, Stanfield S, Corbin D, Helinski DR (1980). Broad host range DNA cloning system for gram-negative bacteria: construction of a gene bank of *Rhizobium meliloti*. Proc Natl Acad Sci U S A.

[12] 1. Ely B. Vectors for transposon mutagenesis of non-enteric bacteria. MGG Mol Gen Genet 1985;200:302–304. doi:10.1007/BF00425440. 10.1007/BF00425440 2993823

[13] Schäfer A, Kalinowski J, Simon R, Seep-Feldhaus AH, Pühler A (1990). High-frequency conjugal plasmid transfer from gram-negative *Escherichia coli* to various gram-positive coryneform bacteria. J Bacteriol.

[14] Rachinger M, Bauch M, Strittmatter A, Bongaerts J, Evers S, Maurer K-H (2013). Size unlimited markerless deletions by a transconjugative plasmid-system in *Bacillus licheniformis*. J Biotechnol.

[15] Tominaga Y, Ohshiro T, Suzuki H (2016). Conjugative plasmid transfer from *Escherichia coli* is a versatile approach for genetic transformation of thermophilic *Bacillus* and *Geobacillus* species. Extremophiles.

[16] Espinosa M, del Solar G, Rojo F, Alonso JC (1995). Plasmid rolling circle replication and its control. FEMS Microbiol Lett.

[17] Lin Y-L, Blaschek HP (1984). Transformation of heat-treated *Clostridium acetobutylicum* protoplasts with pUB110 plasmid DNA. Appl Environ Microbiol.

[18] Johnson CM, Grossman AD (2016). The composition of the cell envelope affects conjugation in B*acillus subtilis*. J Bacteriol.

[19] Westers L, Westers H, Quax WJ (2004). *Bacillus subtilis* as cell factory for pharmaceutical proteins: a biotechnological approach to optimize the host organism. Biochim Biophys Acta.

[20] Jan J, Valle F, Bolivar F, Merino E (2000). Characterization of the 5′ subtilisin (aprE) regulatory region from *Bacillus subtilis*. FEMS Microbiol Lett.

[21] Bien TLT, Tsuji S, Tanaka K, Takenaka S, Yoshida K (2014). Secretion of heterologous thermostable cellulases in *Bacillus subtilis*. J Gen Appl Microbiol.

[22] Ferrari E, Henner DJ, Perego M, Hoch JA (1988). Transcription of *Bacillus subtilis* subtilisin and expression of subtilisin in sporulation mutants. J Bacteriol.

[23] De Man JC, Rogosa M, Sharpe ME (1960). A medium for the cultivation of lactobacilli. J Appl Bacteriol.

[24] Bertani G (1951). Studies on lysogenesis. I. The mode of phage liberation by lysogenic *Escherichia coli*. J Bacteriol.

[25] Gibson DG, Young L, Chuang R-Y, Venter JC, Hutchison CA, Smith HO (2009). Enzymatic assembly of DNA molecules up to several hundred kilobases. Nat Methods.

[26] Wang W, Malcolm BA (1999). Two-stage PCR protocol allowing introduction of multiple mutations, deletions and insertions using QuikChange site-directed mutagenesis. BioTechniques.

[27] Schanzenbach C, Schmidt FC, Breckner P, Teese MG, Langosch D (2017). Identifying ionic interactions within a membrane using BLaTM, a genetic tool to measure homo- and heterotypic transmembrane helix-helix interactions. Sci Rep.

[28] Loy A, Schulz C, Lücker S, Schöpfer-Wendels A, Stoecker K, Baranyi C (2005). 16S rRNA gene-based oligonucleotide microarray for environmental monitoring of the betaproteobacterial order “*Rhodocyclales*”. Appl Environ Microbiol.

[29] Anagnostopoulos C, Spizizen J (1961). Requirements for transformation in *Bacillus subtilis*. J Bacteriol.

[30] Michna RH, Zhu B, Mäder U, Stülke J (2016). SubtiWiki 2.0--an integrated database for the model organism *Bacillus subtilis*. Nucleic Acids Res.

