# Supplementary material for: Transmating: conjugative transfer of a new broad host range expression vector to various Bacillus species using a single protocol
Source: BMC Microbiol. 2018 Jun 8;18:56. doi: 10.1186/s12866-018-1198-4 (PMC5994095; doi:10.1186/s12866-018-1198-4)
Supplement: Supplementary file 1 — Table 1. Selection of well-known and exotic Bacillus species with some of their products and properties (including references). The listed species were also used in this study. (DOCX 20 kb) [file 12866_2018_1198_MOESM1_ESM.docx]

# Additional File 1

## Transmating: Conjugative transfer of a new broad host range expression vector to various *Bacillus* species using a single protocol

Simon Heinze^1^, Petra Kornberger^1,^*, Christian Grätz^1^, Wolfgang H. Schwarz^1^, Vladimir V. Zverlov^1,2^, Wolfgang Liebl^1^

Affiliations:

^1^: Department of Microbiology, Technical University of Munich, Emil-Ramann-Str. 4, D-85354 Freising-Weihenstephan, Germany

^2^: Institute of Molecular Genetics, Russian Academy of Science, Kurchatov Sq. 2, 123182, Moscow, Russia

*: Corresponding author

**Additional table 1: Selection of well-known and exotic Bacillus species with some of their products and properties.**

| Well-known *Bacillus* species with established industrial applications | | |
| --- | --- | --- |
| Species | **Selected products or properties** | **References** |
| *B. subtilis* | - Model organism for Gram-positive bacteria - Important host for the production of industrial enzymes (proteases, amylases, lipases) and pharmaceutical proteins | [1-3] |
| *B. licheniformis* | - Industrial strain (production of thermostable α-amylase and proteases) - Yields of secreted enzymes up to 20-25 g/l | [4, 5] |
| *B. megaterium* | - Recombinant enzymes and therapeutic proteins - Vitamin B_12_, l-lysine, penicillin G acyclase and others | [6, 7] |
| *B. pumilus* | - Alternative to *B. licheniformis* in production of alkaline proteases - Production of xylanases, lipases and keratinases | [5, 8] |
| *Paenibacillus polymyxa* | - Promotion of plant growth and protection against plant pathogens - Production of antimicrobial compounds (e.g. polymyxin) | [9] |
| Less characterized strains with potential applications | | |
| Species | **Selected products or properties** | **References** |
| *B. sonorensis* | - Production of bacteriocins and (hypothermostable) lipases | [10–12] |
| *B. mycoides* | - Production of poly-3-hydroxybutyrate (PHB) or titanium dioxide nanoparticles for solar cells | [13–15] |
| *B. pseudomycoides* | - Phenotypically very similar to *B. mycoides*, but genetically different - Production of pseudomycoicidin (class II lantibiotic active against Gram-positive bacteria) | [16, 17] |
| *B. vallismortis* | - Close relative of *B. subtilis* - Production of an organic-solvent- and thermostable alkalophilic cellulase and a temperature- and pH-stable laccase | [18-20] |
| *B. mojavensis* | - Close relative of *B. subtilis, B. amyloliquefaciens* - Potential applications in food and feed: production of prebiotic galacto-rhamnogalacturonan fibers in piglet feed, production of xylo-oligosaccharides (XOS) from garlic straw, pectinase production for improvement of yield in sesame oil production | [21-24] |

**References**

1. Schumann W. Production of recombinant proteins in *Bacillus subtilis*. Adv Appl Microbiol. 2007;62:137–89. doi:10.1016/S0065-2164(07)62006-1.

2. Westers L, Westers H, Quax WJ. *Bacillus subtilis* as cell factory for pharmaceutical proteins: a biotechnological approach to optimize the host organism. Biochim Biophys Acta. 2004;1694:299–310. doi:10.1016/j.bbamcr.2004.02.011.

3. Wenzel M, Müller A, Siemann-Herzberg M, Altenbuchner J. Self-inducible *Bacillus subtilis* expression system for reliable and inexpensive protein production by high-cell-density fermentation. Appl Environ Microbiol. 2011;77:6419–25.

4. Schallmey M, Singh A, Ward OP. Developments in the use of *Bacillus* species for industrial production. Can J Microbiol. 2004;50:1–17.

5. Küppers T, Steffen V, Hellmuth H, O’Connell T, Bongaerts J, Maurer K-H, et al. Developing a new production host from a blueprint: *Bacillus pumilus* as an industrial enzyme producer. Microb Cell Fact. 2014;13:46. doi:10.1186/1475-2859-13-46.

6. Sharma A, Satyanarayana T. Comparative Genomics of *Bacillus* species and its Relevance in Industrial Microbiology. Genomics Insights. 2013;6:25–36. doi:10.4137/GEI.S12732.

7. Korneli C, David F, Biedendieck R, Jahn D, Wittmann C. Getting the big beast to work-Systems biotechnology of *Bacillus megaterium* for novel high-value proteins. J Biotechnol. 2013;163:87–96. doi:10.1016/j.jbiotec.2012.06.018.

8. Wang C, Yu S, Song T, He T, Shao H, Wang H. Extracellular Proteome Profiling of *Bacillus pumilus* SCU11 Producing Alkaline Protease for Dehairing. J Microbiol Biotechnol. 2016;26:1993–2005. doi:10.4014/jmb.1602.02042.

9. Grady EN, MacDonald J, Liu L, Richman A, Yuan Z-C. Current knowledge and perspectives of *Paenibacillus*: a review. Microb Cell Fact. 2016;15:203. doi:10.1186/s12934-016-0603-7.

10. Chopra L, Singh G, Jena KK, Verma H, Sahoo DK. Bioprocess development for the production of sonorensin by *Bacillus sonorensis* MT93 and its application as a food preservative. Bioresour Technol. 2015;175:358–66. doi:10.1016/j.biortech.2014.10.105.

11. Bhosale H, Shaheen U, Kadam T. Characterization of a Hyperthermostable Alkaline Lipase from *Bacillus sonorensis* 4R. Enzyme Res. 2016;2016:1–11. doi:10.1155/2016/4170684.

12. Nerurkar M, Joshi M, Adivarekar R. Bioscouring of Cotton using Lipase from Marine Bacteria *Bacillus sonorensis*. Appl Biochem Biotechnol. 2015;175:253–65. doi:10.1007/s12010-014-1259-6.

13. Thakur PS, Borah B, Baruah SD, Nigam JN. Growth-associated production of poly-3-hydroxybutyrate by *Bacillus mycoides*. Folia Microbiol (Praha). 2001;46:488–94. doi:10.1007/BF02817991.

14. Borah B, Thakur PS, Nigam JN. The influence of nutritional and environmental conditions on the accumulation of poly-beta-hydroxybutyrate in *Bacillus mycoides* RLJ B-017. J Appl Microbiol. 2002;92:776–83. doi:10.1046/j.1365-2672.2002.01590.x.

15. Órdenes-Aenishanslins NA, Saona LA, Durán-Toro VM, Monrás JP, Bravo DM, Pérez-Donoso JM. Use of titanium dioxide nanoparticles biosynthesized by *Bacillus mycoides* in quantum dot sensitized solar cells. Microb Cell Fact. 2014;13:90. doi:10.1186/s12934-014-0090-7.

16. Nakamura LK. *Bacillus pseudomycoides* sp. nov. Int J Syst Bacteriol. 1998;48:1031–5. doi:10.1099/00207713-48-3-1031.

17. Basi-Chipalu S, Dischinger J, Josten M, Szekat C, Zweynert A, Sahl H-G, et al. Pseudomycoicidin, a Class II Lantibiotic from *Bacillus pseudomycoides*. Appl Environ Microbiol. 2015;81:3419–29. doi:10.1128/AEM.00299-15.

18. Roberts MS, Nakamura LK, Cohan FM. *Bacillus vallismortis* sp. nov., a Close Relative of *Bacillus subtilis*, Isolated from Soil in Death Valley, California. Int J Syst Bacteriol. 1996;46:470–5. doi:10.1099/00207713-46-2-470.

19. Gaur R, Tiwari S. Isolation, production, purification and characterization of an organic-solvent-thermostable alkalophilic cellulase from *Bacillus vallismortis* RG-07. BMC Biotechnol. 2015;15:19. doi:10.1186/s12896-015-0129-9.

20. Zhang C, Zhang S, Diao H, Zhao H, Zhu X, Lu F, et al. Purification and Characterization of a Temperature- and pH-Stable Laccase from the Spores of *Bacillus vallismortis* fmb-103 and Its Application in the Degradation of Malachite Green. J Agric Food Chem. 2013;61:5468–73. doi:10.1021/jf4010498.

21. Roberts MS, Nakamura LK, Cohan FM. *Bacillus mojavensis* sp. nov., Distinguishable from *Bacillus subtilis* by Sexual Isolation, Divergence in DNA Sequence, and Differences in Fatty Acid Composition. Int J Syst Bacteriol. 1994;44:256–64. doi:10.1099/00207713-44-2-256.

22. Jers C, Strube ML, Cantor MD, Nielsen BKK, Sørensen OB, Boye M, et al. Selection of *Bacillus* species for targeted in situ release of prebiotic galacto-rhamnogalacturonan from potato pulp in piglets. Appl Microbiol Biotechnol. 2017;101:3605–15. doi:10.1007/s00253-017-8176-x.

23. Kallel F, Driss D, Chaabouni SE, Ghorbel R. Biological Activities of Xylooligosaccharides Generated from Garlic Straw Xylan by Purified Xylanase from *Bacillus mojavensis* UEB-FK. Appl Biochem Biotechnol. 2015;175:950–64. doi:10.1007/s12010-014-1308-1.

24. Ghazala I, Sayari N, Romdhane M Ben, Ellouz-Chaabouni S, Haddar A. Assessment of pectinase production by *Bacillus mojavensis* I4 using an economical substrate and its potential application in oil sesame extraction. J Food Sci Technol. 2015;52:7710–22. doi:10.1007/s13197-015-1964-3.
