# Supplementary material for: Transmating: conjugative transfer of a new broad host range expression vector to various Bacillus species using a single protocol
Source: BMC Microbiol. 2018 Jun 8;18:56. doi: 10.1186/s12866-018-1198-4 (PMC5994095; doi:10.1186/s12866-018-1198-4)
Supplement: Supplementary file 2 — Table 2. Strains used in this study and determination of MIC of Kan or Pol (including references). (DOCX 19 kb) [file 12866_2018_1198_MOESM2_ESM.docx]

# Additional File 2

## Transmating: Conjugative transfer of a new broad host range expression vector to various *Bacillus* species using a single protocol

Simon Heinze^1^, Petra Kornberger^1,^*, Christian Grätz^1^, Wolfgang H. Schwarz^1^, Vladimir V. Zverlov^1,2^, Wolfgang Liebl^1^

Affiliations:

^1^: Department of Microbiology, Technical University of Munich, Emil-Ramann-Str. 4, D-85354 Freising-Weihenstephan, Germany

^2^: Institute of Molecular Genetics, Russian Academy of Science, Kurchatov Sq. 2, 123182, Moscow, Russia

*: Corresponding author

**Additional Table 2: Strains used in this study and determination of MIC of Kan or Pol.**

| **Strains resistant to 40 µg/ml polymyxin B and sensitive to 10 µg/ml kanamycin** | | | | | | | |
| --- | --- | --- | --- | --- | --- | --- | --- |
| **Species** | **Source** |  |  |  |  |  |  |
| ***B. licheniformis* DSM13 ^T^** | DSMZ |  | | | | |  |
| ***B. pumilus* DSM27** **^T^** | DSMZ |  | | | | |  |
| ***B. sonorensis* DSM13779 ^T^** | DSMZ |  | | | | |  |
| ***P. polymyxa* DSM356** | DSMZ |  | | | | |  |
| **Growth of strains resistant to 40 µg/ml polymyxin B at different kanamycin concentrations** | | | | | | | |
| **Species** | **Source** | **Kanamycin concentration [µg/ml]** | | | | | |
|  |  | **12.5** | **25** | **50** | **100** | **200** | |
| ***B. mycoides* DSM2048** **^T^** | DSMZ | +++ | ++ | - | - | - | |
| ***B. megaterium* DSM32 ^T^** | DSMZ | + | + | - | - | - | |
| *B. thuringiensis* DSM2046 ^T^ | DSMZ | +++ | ++ | + | + | + | |
| *B. amyloliquefaciens* DSM7^T^ | DSMZ | +++ | + | + | + | + | |
| *B. pocheonensis* DSM18135 ^T^ | DSMZ | +++ | ++ | + | + | - | |
| ***B. pseudomycoides* DSM12442** **^T^** | DSMZ | + | - | - | - | - | |
| **Growth of strains sensitive to 10 µg/ml kanamycin at different polymyxin B concentrations** | | | | | | | |
| **Species** | **Source** | **Polymyxin B concentration [µg/ml]** | | | | | |
|  |  | **2.5** | **5** | **10** | **20** | **40** | |
| *B. badius* DSM23 ^T^ | DSMZ | - | - | - | - | - | |
| *B. bataviensis* WS4576 | TUM-ME | - | - | - | - | - | |
| *B. cohnii* DSM6307 ^T^ | DSMZ | - | - | - | - | - | |
| *B. flexus* DSM1320 ^T^ | DSMZ | - | - | - | - | - | |
| *B. foraminis* DSM19613 ^T^ | DSMZ | - | - | - | - | - | |
| *B. ginsengihumi* WS8095 | TUM-ME | - | - | - | - | - | |
| *B. horikoshii* WS2157 | TUM-ME | - | - | - | - | - | |
| ***B. mojavensis* DSM9205** **^T^** | DSMZ | +++ | +++ | +++ | - | - | |
| *B. niabensis* WS9147 | TUM-ME | - | - | - | - | - | |
| *B. niacini* WS4575 | TUM-ME | - | - | - | - | - | |
| ***B. oleronius* WS8036** | TUM-ME | -/++ * | - | - | - | - | |
| ***B. subtilis* RIK1285** | Takara-Bio Inc., Kusatsu, Japan | +++ | +++ | - | - | - | |
| ***B. vallismortis* DSM11031 ^T^** | DSMZ | +++ | +++ | +++ | ++ | - | |
| *Fictibacillus arsenicus WS4538* | TUM-ME | - | - | - | - | - | |
| **Verification of polymyxin B sensitivity of used *E. coli* strains** | | | | | | | |
| **Strain** | **Source or reference** |  |  |  |  |  |  |
| *E. coli* TOP10 | Life technologies, Carlsbad, USA | - | - | - | - | - | |
| *E. coli* HB101 pRK2013 | [1, 2] | - | - | - | - | - | |

The minimal inhibitory concentrations were determined by serial dilution tests. Bacterial strains written in **bold face** were selected as acceptor strains for transmating experiments. Type strains are indicated by a superscript ^T^. Legend: +++: strong growth within 24 h, ++ medium growth within 24 h, +: weak but detectable growth within 24 h, - : no growth detectable within 24 h. *: medium growth detectable after incubation for 48 h. DSMZ: Leibniz-Institut DSMZ-Deutsche Sammlung von Mikroorganismen und Zellkulturen GmbH, Braunschweig, Germany; TUM-ME: Chair for Microbial Ecology, Department of Biosciences, Technical University of Munich, Freising, Germany

**References**

1. Figurski DH, Helinski DR. Replication of an origin-containing derivative of plasmid RK2 dependent on a plasmid function provided in trans. Proc Natl Acad Sci U S A. 1979;76:1648–52.

2. Ditta G, Stanfield S, Corbin D, Helinski DR. Broad host range DNA cloning system for gram-negative bacteria: construction of a gene bank of *Rhizobium meliloti*. Proc Natl Acad Sci U S A. 1980;77:7347–51.
